# Treatment Algorithm of Peripancreatic Arteries Aneurysm Coexisting with Coeliac Artery Lesion: Single Institution Experience

**DOI:** 10.1155/2018/5745271

**Published:** 2018-07-18

**Authors:** Robert Antoniak, Laretta Grabowska-Derlatka, Rafał Maciąg, Tomasz Ostrowski, Ireneusz Nawrot, Zbigniew Gałązka, Sławomir Nazarewski, Olgierd Rowiński

**Affiliations:** ^1^2nd Department of Radiology, Medical University of Warsaw, Banacha 1a st., 02-097 Warsaw, Poland; ^2^Department of General and Endocrine Surgery, Medical University of Warsaw, Banacha 1a st., 02-097 Warsaw, Poland; ^3^Department of General, Vascular and Transplant Surgery, Medical University of Warsaw, Banacha 1a st., 02-097 Warsaw, Poland

## Abstract

**Introduction:**

True aneurysms of peripancreatic arterial arcades (PAAAs) coexisting with celiac axis lesion are often asymptomatic. However, they may rupture regardless of their size and cause life-threatening hypovolemia. No treatment guidelines exist to date. We present a series of 21 patients and our management algorithm.

**Material and Methods:**

For ruptured aneurysms we preformed endovascular embolization. Further treatment was dependent on patient's condition and control studies. In case of unruptured aneurysms, we assessed collateral circulation between superior mesenteric artery and celiac axis in angio-CT. If there was a pathway free from aneurysms, endovascular approach was chosen. Otherwise, surgical or combined treatment was favored.

**Results:**

Endovascular treatment was performed in 14 patients with no complications. Follow-up studies revealed incomplete occlusion of the aneurysms in two cases. Surgical or combined treatment was performed in 7 patients with three serious perioperative complications. They were managed conservatively in two cases and surgically in one. Follow-up studies showed aneurismal dilatation and stenosis of a renohepatic by-pass in one case.

**Conclusion:**

We present our management algorithm of PAAAs. Our results support the leading role of endovascular treatment. We present its limitations favoring surgical or combined treatment. All patients should be carefully followed.

## 1. Introduction

True aneurysms of peripancreatic arterial arcades (PAAAs) coexisting with celiac axis stenosis or occlusion are rare and often found incidentally. Limited flow through celiac trunk is compensated by collateral circulation from superior mesenteric artery through peripancreatic arteries. This increased flow is responsible for enlargement of these arteries and aneurysm formation. Natural course of PAAAs is unknown. They are prone to rupture and there are no risk factors for their rupture. Thus, every aneurysm should be considered for treatment. To date no treatment guidelines exist. However, endovascular embolization emerges as the treatment of choice. The main concern of treatment is maintenance of hepatic perfusion. Unintentional closure of peripancreatic artery may cause hepatic ischemia in case of insufficient collateral circulation. We present a series of 21 patients with PAAAs treated using endovascular, surgical, and combined techniques and propose management algorithm.

## 2. Material and Methods

**Figure 1:**
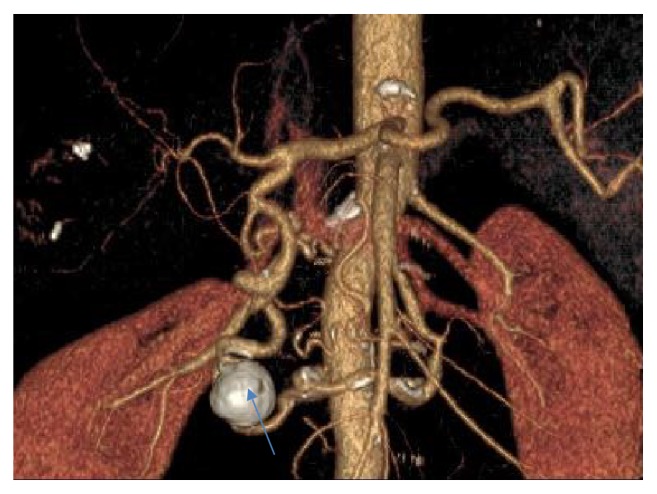
VR reconstruction of peripancreatic arteries in patient with PAAA and a collateral pathway between celiac axis and superior mesenteric artery free from aneurysm.

**Figure 2:**
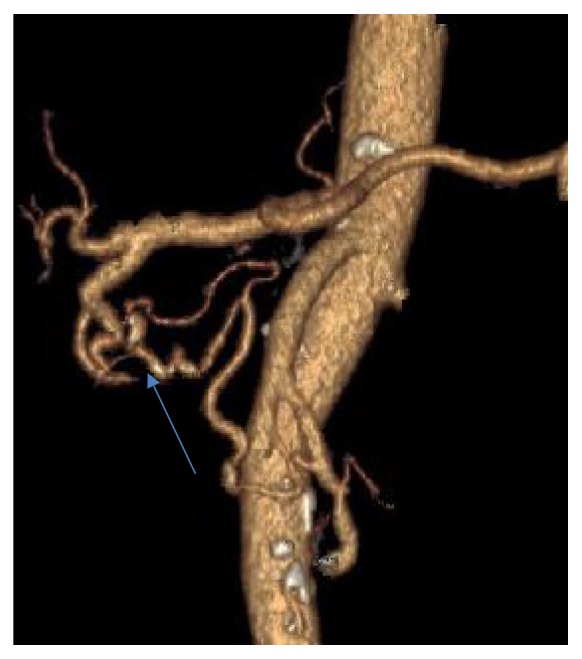
Detailed view of a collateral pathway free from aneurysm, the same patient as Figure 1.

**Figure 3:**
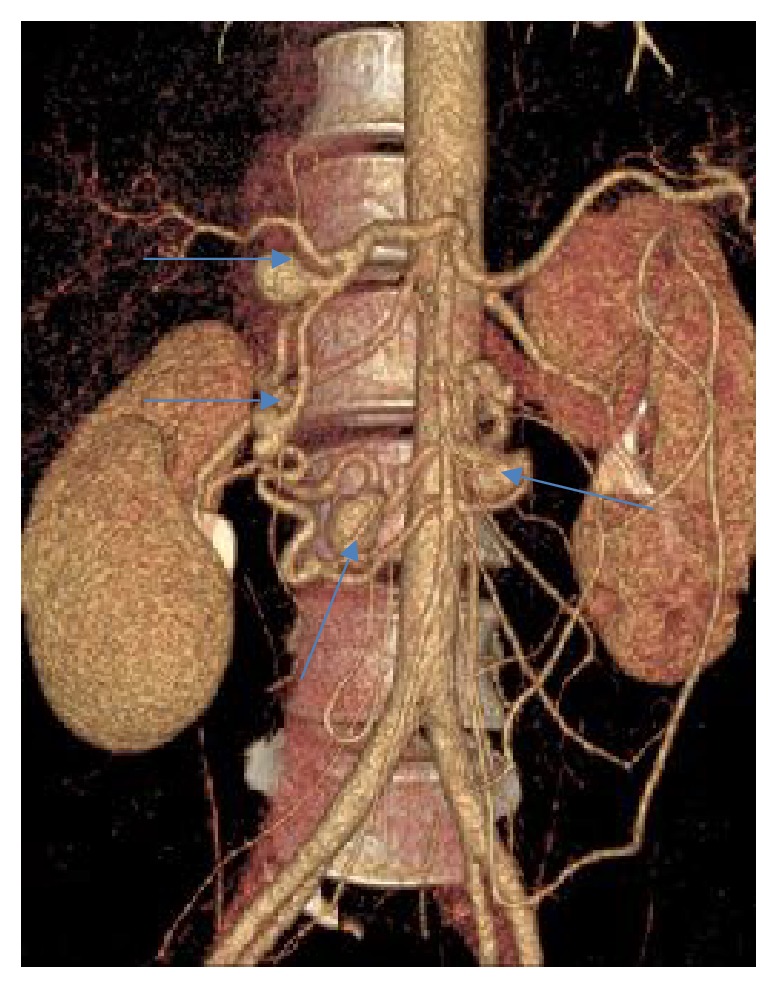
VR reconstruction of peripancreatic arteries in patient with multiple PAAAs and no collateral pathway between celiac axis and superior mesenteric artery free from aneurysm.

**Figure 4:**
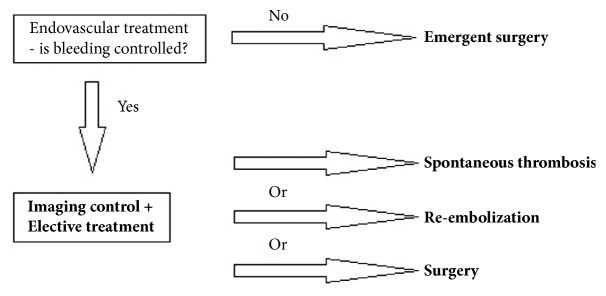
Management algorithm of ruptured PAAA.

**Figure 5:**
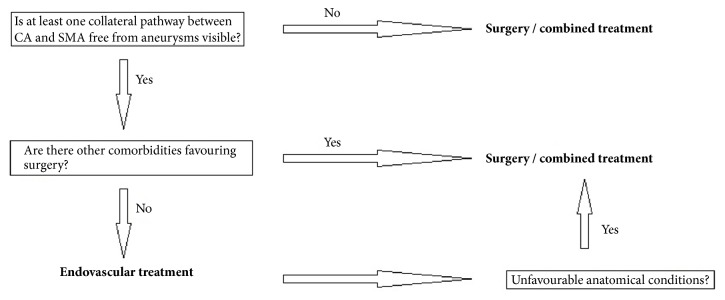
Management algorithm of unruptured PAAA.

Endovascular embolization was first-choice treatment of both ruptured and unruptured PAAAs. It allowed control of bleeding and gave time to plan further elective treatment in emergent situation. On the other hand, it was usually definitive treatment for stable aneurysms. Some patients were unsuitable for embolization. We tried to identify them based on prior angio-CT studies. Therefore, we evaluated four different collateral pathways connecting celiac trunk and superior mesenteric artery circulations (anterior pancreaticoduodenal arteries, posterior pancreaticoduodenal arteries, dorsal pancreatic artery, and gastroepiploic arteries). Patients, who had at least one visible collateral pathway free from aneurysms (group I, Figures 1 and 2), were treated with endovascular techniques. This collateral pathway would act as a safety valve maintaining hepatic perfusion in case of closure of other routes affected by aneurysms. Thus, these patients were thought to have lower risk of hepatic ischemia complicating treatment. On the other hand, high-risk patients (group II, Figure 3) had aneurysms involving every visible collateral pathway. They were treated primarily with surgical revascularization. Aneurysms of peripancreatic arteries were either simultaneously resected (subgroup IIa) or embolized in a separate procedure (subgroup IIb) depending on surgeon's decision. The management algorithm is summarized in Figures 4 and 5.

Other, coexisting diseases also influenced treatment approach. Patients treated only surgically were followed with CT. Those who underwent embolization were preferentially followed using angiography and/or MRI.

Our group of patients consisted of 3 men and 18 women with a mean age of 55,6 years (range, 26 to 84). The diagnosis of PAAAs was established in angio-CT studies in all cases. One patient presented with ruptured aneurysm and underwent emergent endovascular embolization. The other 20 patients were diagnosed with PAAAs either by chance or due to nonspecific abdominal complaints. Basing on analysis of peripancreatic circulation, 14 patients would have been classified into group I and 6 into group II. However, one patient suffered coexisting pancreatic head cancer requiring pancreaticoduodenectomy and was reclassified into group II. Finally, group I consisted of 13 patients and group II of 7. Group I patients underwent endovascular treatment in the radiology suite on an angiography system (Axiom Artis, Siemens Medical Solutions, Erlangen, Germany). First, diagnostic angiography of superior mesenteric artery was performed using Cobra or SIM1 catheter (Cook Group Inc., Bloomington, USA) to confirm presence of a collateral pathway free from aneurysms. Then, the aneurysm was cannulated with a microcatheter and embolized with detachable coils (using either Rebar 18 catheter and Concerto coils, Covidien, Irvine, CA, USA, or PX Slim catheter and Ruby coils, Penumbra, Alameda, CA, USA). If a stent assisted coiling was required, long sheath was introduced (Flexor, Cook Group Inc., Bloomington, USA, or Destination, Terumo Corporation, Somerset, USA) to assure better stability. Stent (XPERT, Abbott Vascular, Santa Clara, CA, USA, or Astron Pulsar, Biotronik, SE & Co. KG, Berlin, Germany) was placed in the parent artery to bridge the aneurysm neck. Detachable coils were then introduced to the aneurysm via stent mesh. Group I patients were followed with angiography and/or MRI 3-6 months after the procedure and then early.

In subgroup IIa, five patients were treated with surgical revascularization with simultaneous aneurysms resection. The revascularization procedures consisted of renohepatic by-pass in three cases and aortohepatic by-pass and end-to-end anastomosis of gastroduodenal artery in one case each. These patients were followed with CT in a similar time schedule as group I. In subgroup IIb two patients underwent surgical procedures followed by endovascular embolization of PAAAs. Different surgical approaches were used depending on individual patients' factors. The patient with pancreatic cancer underwent pancreaticoduodenectomy with aortohepatic by-passing. Subsequently, embolization of one PAAA was performed. He was followed only with CT. Another patient presented with 6 PAAAs. She underwent aortohepatic by-passing and two-step embolization of the aneurysms. She is still waiting for a follow-up study.

## 3. Results

One patient underwent emergent treatment of a ruptured aneurysm. Successful embolization of the bleeding aneurysm was achieved. The patient was discharged in a good condition and is waiting for a follow-up study.

**Figure 6:**
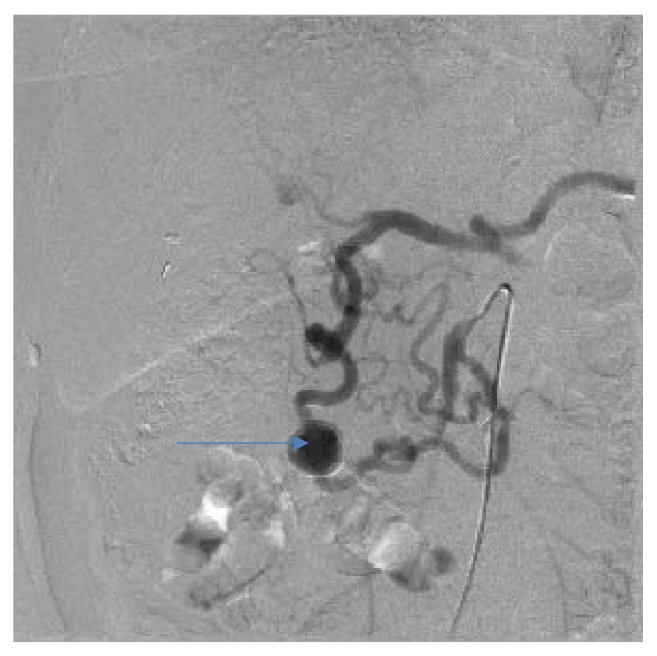
Angiographic view of peripancreatic arteries with PAAA before treatment, the same patient as Figures 1 and 2.

**Figure 7:**
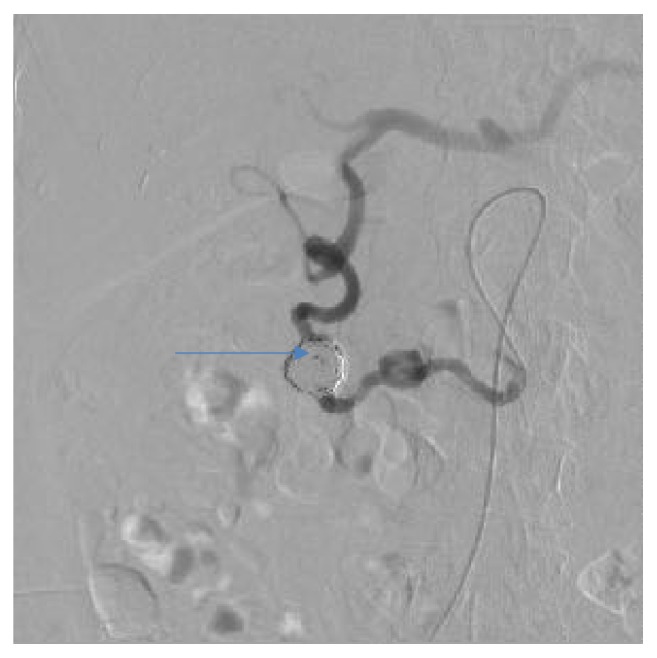
Angiographic view after embolization, the same patient as Figure 4.

**Figure 8:**
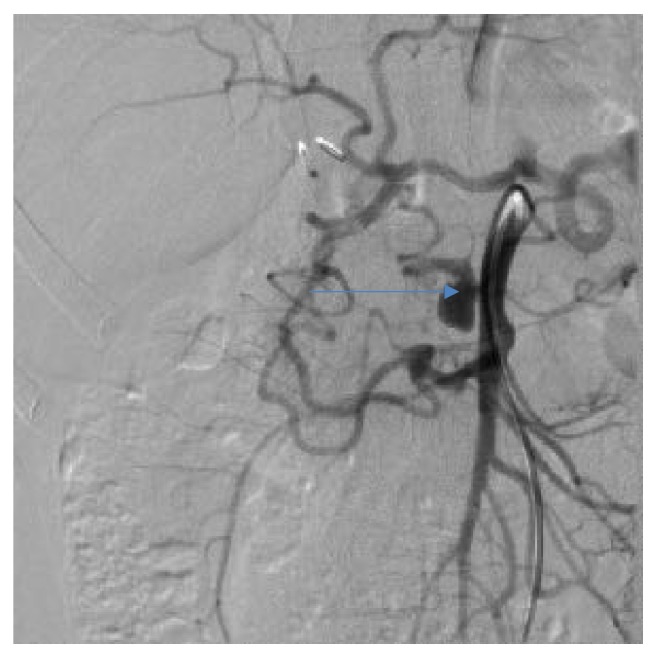
Angiographic view of peripancreatic arteries with PAAA before treatment, another patient.

**Figure 9:**
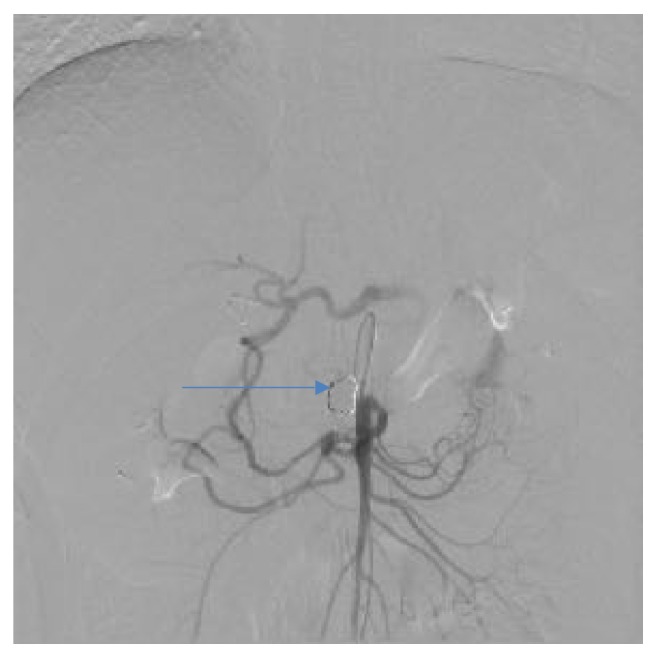
Angiographic view after embolization, the same patient as Figure 8.

**Figure 10:**
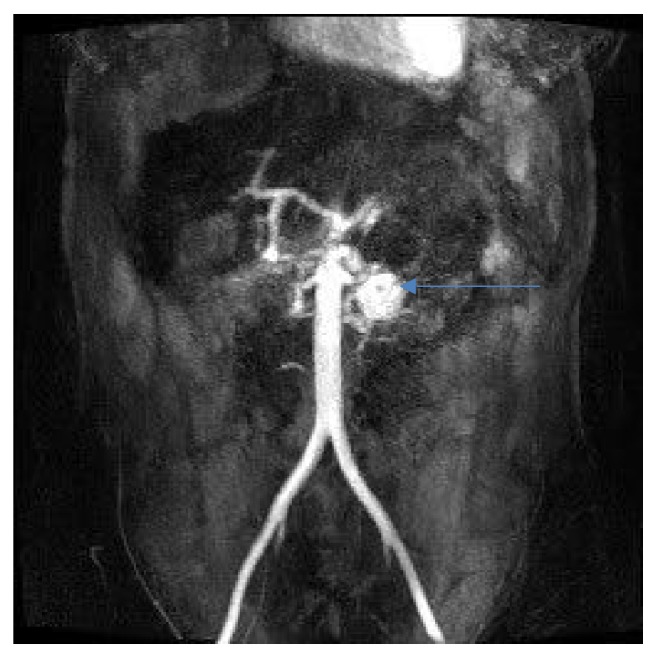
Follow-up MRI angiography showing recanalization of PAAA.

**Figure 11:**
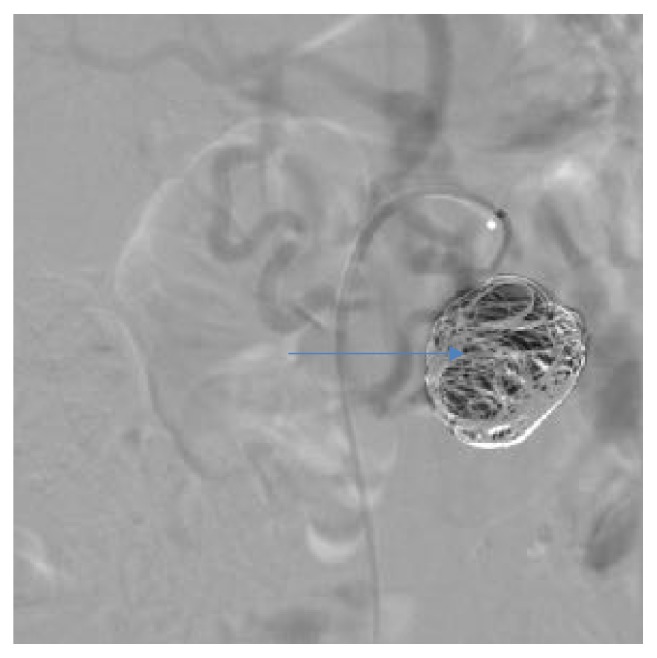
Follow-up angiography showing recanalization of PAAA, the same patient as Figure 10.

**Figure 12:**
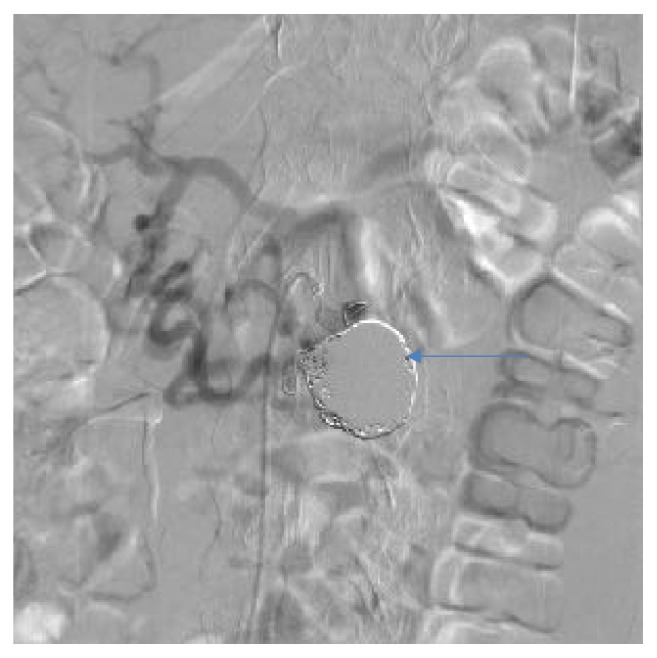
Angiographic view after reembolization showing complete occlusion of PAAA, the same patient as Figure 11.

Group I patients were treated with endovascular techniques (Figures 6, 7, 8, and 9). There were no complications in the periprocedural period. Unfortunately, one patient was lost to follow-up. Two other patients are waiting for their follow-up studies. Ten patients were followed with a mean follow-up time of 13,3 months (range, 3 to 29). The follow-up studies of choice were angiography and/or MRI. Angio-CT was performed in three cases due to unrelated comorbidities. Follow-up studies revealed residual filling of the aneurysm with contrast media in two cases (Figures 10 and 11). Additional embolization was performed with immediate successful results achieved in both cases (Figure 12). In one patient it was confirmed after 9 months with both angiography and MRI while the other patient is still waiting for a follow-up study. All other aneurysms remained stable in size with no filling of contrast media.

**Figure 13:**
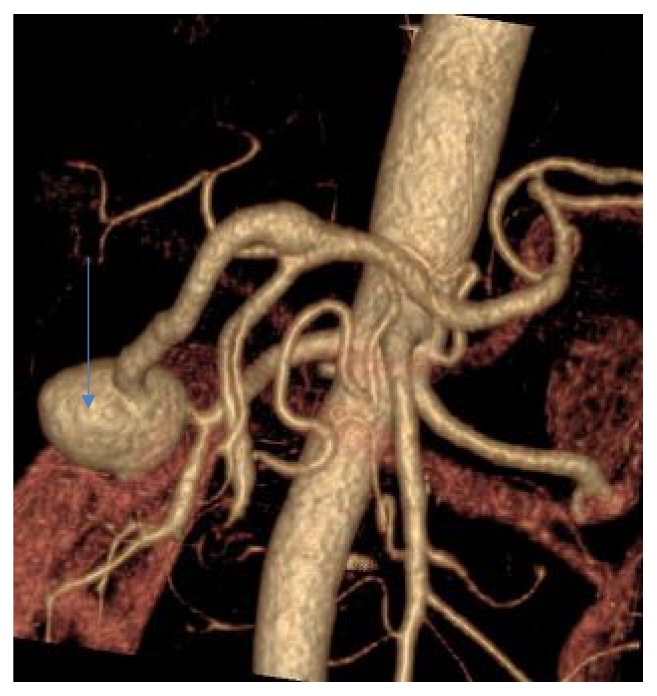
VR reconstruction showing stenosis and aneurismal dilatation of renohepatic by-pass.

Group II patients were treated surgically. In subgroup IIa aneurysms were simultaneously resected. There were two serious complications of treatment in this subgroup. One patient suffered infection of aortohepatic by-pass, splenic infarction, and insignificant pulmonary embolism, while the other suffered injury of right hepatic artery and common bile duct. These complications were managed conservatively. Both patients were discharged in good condition but were lost to follow-up. The other three patients underwent renohepatic by-passing and were followed with CT. One by-pass remained stable in a 149 months' follow-up and another dilated minimally in 92 months' follow-up. Stenosis and aneurismal dilatation of the graft occurred in one case (Figure 13) and required several endovascular procedures. Eventually, the graft was closed with a covered stent after separate collateral pathway between superior mesenteric artery and celiac trunk had developed.

In subgroup IIb surgical revascularization was followed by endovascular embolization. The patient suffering pancreatic cancer underwent pancreaticoduodenectomy, which was complicated by arterial bleeding, fluid collections, and biliary fistula. These complications were successfully managed in subsequent surgical procedures. Aneurysm's size remained stable 8 months after embolization in follow-up CT scans. The other patient, diagnosed with 6 aneurysms, underwent successful aortohepatic by-passing and two-step embolization of the aneurysms with no complications and is still waiting for a follow-up study.

No new aneurysms developed in the observation period.

## 4. Discussion

PAAAs are often found by chance in patients undergoing imaging studies for other reasons. However, they are prone to rupture, which is unpredictable and potentially life threatening. The mortality rate of ruptured PAAAs reaches 21% [1]. Size and multiplicity of the aneurysms do not correlate with risk of their rupture [1, 2]. Several treatment options are described in the literature. Endovascular approach emerges as the treatment of choice in both ruptured and unruptured aneurysms [3, 4]. Previous publications proved its effectiveness with extremely low mortality and morbidity rates, lower compared to surgery [5, 6]. The main techniques are coil packing and aneurysm isolation [7]. Some authors recommend simultaneous treatment of celiac axis lesion with angioplasty and stenting. Interestingly, restoration of celiac trunk patency alone may result in blood stagnation in peripancreatic arteries promoting aneurysm thrombosis [8]. Surgical procedures gradually lose their significance with advancement of endovascular approach. Surgical treatment may take form of resection or ligation of the aneurysms with or without arterial reconstruction [9, 10]. Pancreaticoduodenectomy is rarely performed. Combined treatment with surgical by-passing and endovascular embolization may be required in difficult cases [11]. To date, there are no guidelines concerning management of PAAAs. The most appropriate approach is chosen depending on urgency of the procedure, surgeon's experience, and local preferences.

We present our treatment algorithm of PAAAs. Endovascular procedures were the treatment of choice in our institution. We performed urgent embolization in patients with ruptured aneurysms. Surgery is particularly challenging in emergent situation due to retroperitoneal location of the aneurysms, their proximity to pancreas, and presence of a hematoma. Embolization proved to be efficient in this setting. Even when the procedure is not immediately successful, it allows control of the bleeding. In some cases complete thrombosis of the aneurysm occurs spontaneously. If the residual flow persists, reembolization or surgery may be performed electively. In our series only one patient presented with ruptured aneurysm and urgent embolization was successful. Other authors confirmed usefulness of endovascular procedures in emergent situation [12, 13]. Suzuki et al. [12] reported series of seven patients with ruptured aneurysms. Complete embolization was achieved in four cases. Residual flow remained in the other three cases, but bleeding was controlled. Follow-up studies showed spontaneous, complete thrombosis of these aneurysms. Corey et al. [13] reported series of 35 patients, seven of whom presented with ruptured aneurysms. Embolization was immediately successful in all cases, but surgical ligation was required in two cases in the postprocedural period due to rebleeding. Authors reported morbidity rate of 50% and mortality rate of 14,3% at 30 days after urgent treatment.

Our management strategy of patients with unruptured PAAAs was based on the analysis of peripancreatic arteries connecting celiac trunk and superior mesenteric artery circulations in angio-CT studies. All patients with at least one collateral pathway free from aneurysms underwent angiography and, in favorable anatomical circumstances, were treated with embolization. Results of this treatment were successful with only two patients requiring additional embolization due to contrast filling of the aneurysm in the follow-up study. There were no serious complications in this group. Other authors achieved similar results [14, 15]. We recommend MRI in follow-up studies. In our experience it is at least as sensitive as angiography in evaluating efficacy of embolization, less invasive, and free from radiation exposure.

On the other hand, some patients are unsuitable for this treatment. Lack of collateral pathway free from aneurysms raises a high concern about hepatic perfusion after treatment. Unfavorable anatomical conditions may preclude endovascular embolization. Additionally, other unrelated diseases may require surgical approach, like the patient with pancreatic cancer in our series. All these factors promote primary surgical procedures. Aneurysms are either simultaneously resected or endovascularly treated in a separate procedure. Surgical by-passes require careful follow-up with CT as they are prone to stenoses and dilatations. Results of these strategies were less favorable in our series. Surgical procedures were performed by a team of experienced vascular surgeons. Nevertheless, three serious periprocedural complications occurred. Higher rate of complications can be attributed to patients' anatomical anomalies and coexisting morbidities. Further surgical treatment was required only for complications following pancreaticoduodenectomy, which is a known, high risk procedure. All complications were managed successfully. It should be underlined that the small number of patients makes this group unrepresentative.

There is no clear consensus regarding treatment of celiac trunk lesion in patients with PAAAs. The need for celiac axis revascularization was recently questioned [16]. In the literature there are no cases of aneurysm recurrence after treatment [3, 17]. In our study no new aneurysm developed in the follow-up period. We state that celiac revascularization might not be necessary if the aneurysms can be safely treated with maintenance of hepatic circulation. It should be considered only in symptomatic patients. It is worth noticing that great majority of celiac axis lesions were secondary to median arcuate ligament compression in our series. We do not recommend stenting in this scenario as we have seen stent reocclusions and fractures.

## 5. Conclusion

Advantages of endovascular over surgical treatment of visceral arteries aneurysms with lower mortality and morbidity rates are well documented. It has also been shown in publications concerning management of PAAAs with celiac axis lesion. Our results support the leading role of endovascular treatment in both ruptured and unruptured aneurysms. We also present its limitations and the need for surgical or combined treatment. Our management algorithm is summarized in Figures 4 and 5. All patients should be carefully followed. We recommend MRI in patients treated endovascularly and CT for those treated surgically.

## Data Availability

The data used to support the findings of this study are available from the corresponding author upon request.
